# Effect of Carbon Fiber and Potassium Titanate Whisker on the Mechanical and Impact Tribological Properties of Fe-Based Impregnated Diamond Bit Matrix

**DOI:** 10.3390/ma17112645

**Published:** 2024-05-30

**Authors:** Zhiming Wang, Chengkai Guan, Wucheng Sun, Songcheng Tan, Longchen Duan, Xiaohong Fang

**Affiliations:** 1 Faculty of Engineering, China University of Geosciences, Wuhan 430074, China; 2 National Center for International Research on Deep Earth Drilling and Resource Development, Wuhan 430074, China

**Keywords:** impregnated diamond bit, carbon fiber, potassium titanate whisker, impact wear resistance

## Abstract

Various contents of carbon fibers (CFs) and potassium titanate whiskers (PTWs) were added to an Fe-based impregnated diamond bit (IDB) matrix to enhance its adaptability to percussive–rotary drilling. A series of mechanical tests were conducted successively to find the effects of the reinforcing materials on the properties of the Fe-based IDB samples. Then, the fracture surfaces of the samples were analyzed via scanning electron microscopy (SEM) and energy-dispersive spectroscopy, and the worn surfaces and abrasive debris of the samples were analyzed using a laser scanning confocal microscope and SEM. The results show that both the CF and PTW can effectively improve the hardness and bending strength of an Fe-based IDB matrix, and those parameters reached their maximum values at the additive amount of 1 wt%. However, the CF had a better enhancement effect than the PTW. Furthermore, the CF improved the impact wear resistance of the IDB matrix, with a minimum wear rate of 2.38 g/min at the additive amount of 2 wt%. However, the PTW continuously weakened the impact wear resistance of the IDB matrix with increases in its content. Moreover, the morphologies of the worn surfaces indicated that the minimum roughness of the CF-reinforced IDB matrix decreased significantly to as low as 4.91 μm, which was 46.16% lower than that without CF, whereas the minimum roughness of the PTW-reinforced samples decreased by 11.31%. Meanwhile, the abrasive debris of the CF-reinforced samples was more uniform and continuous compared to that of the PTW-reinforced samples. Overall, the appropriate addition of CF or PTWs can enhance the mechanical properties of Fe-based IDB matrices, which can be used on different formations based on their impact wear resistance.

## 1. Introduction

With the gradual depletion of the Earth’s shallow mineral resources, deep drilling is essential to ensure their supply [1]. Relative to conventional drilling, deep hole drilling faces greater challenges, and hard and complex formations are a major challenge [2,3,4,5]. Impregnated diamond bits (IDBs), due to their unique rock-breaking method, have become the main types of drill bits for deep core drilling. They play a major role in deep and ultra-deep hole drilling in conjunction with wireline coring and hydraulic percussive–rotary drilling techniques [6]. In percussive–rotary drilling, the diamond bit at the bottom of the hole suffers more severe conditions than it in conventional rotary drilling, because it faces multiple loads, such as drilling pressure, drilling torque, and impact force, which raises the performance requirements of IDBs [7].

The cutting efficiency and service life of an IDB are two determinants of the drilling efficiency [8], and the normal wear of the bit matrix is a prerequisite for the successful application of IDBs. If the matrix’s hardness is high and the rock’s abrasiveness is low, the matrix will not be able to wear out in advance, and the diamond will not be exposed normally. Therefore, the IDB matrix’s properties should match the formation’s abrasiveness to better expose the diamond. Because the cutting elements of an IDB are composed of diamond particles and a matrix, the wear of the IDB includes the wear of diamonds, as well as the wear of the bit matrix. Wang [9] classified the wear forms of diamonds in diamond saw blades into eight types and determined that the wear process of diamonds involves a combination of two-body wear and three-body wear. To obtain a normal exposure of diamonds, the bit matrix should wear down at a rate appropriate to the diamond parameters. Thus, the wear resistance of a diamond bit matrix is very important. Many metal composites have high wear resistance, such as some tungsten carbide (WC)-based composites, which often have relatively high hardness [10]. Traditional diamond bit matrices are mainly WC–Co-based, but in recent years, more and more researchers have started to test relatively inexpensive Fe pre-alloyed powders to replace them [11,12,13,14]. With their lower melting points and excellent mechanical properties, pre-alloyed materials cause less thermal damage to diamonds and can ensure the performance of diamond composite materials [15,16].

In addition to particle reinforcement and alloying, fibers are often used to strengthen composite material [17,18,19]. Fibers are widely used as reinforcements in friction materials, building materials, refractory materials, and high-temperature filter fabrics [20,21,22,23]. In composite materials, fiber materials serve as reinforcements to withstand loads during matrix cracking, maintaining the matrix integrity and improving its damage resistance [24,25,26]. It has been found that carbon fibers (CFs) can improve the tensile strength and impact resistance of composites [26,27,28,29,30]. Fiber reinforcement might be a feasible method used to adapt to the impact load on the matrix under percussion and rotation conditions. So far, there has been little research on fiber reinforcement in the field of IDBs. Some researchers have added basalt fibers as additives to the drill bit matrix and found that a small amount of basalt fiber can enhance the mechanical properties of the matrix, but it lowers the wear resistance of the composite material [31,32]. Potassium titanate whiskers (PTWs), a type of ultra-short fiber, are widely used in ceramic brake pads and other friction materials [33,34,35]. They can effectively improve composites’ wear resistance and strengthen their mechanical properties, making them a good reinforcing material [36,37].

Therefore, in this study, CFs and PTWs were added to an Fe-based IDB matrix, and the effects of their different contents on the mechanical and impact tribological properties of the IDB matrix were studied.

## 2. Materials and Methods

### 2.1. Materials and Sample Preparation Methods

The matrix of the IDB comprised an Fe-based pre-alloyed powder FJT-A2 (Hunan Metallurgy Material Institute) of which 70% was iron and 30% was copper. Two types of toughening materials, CF (45 to 55 μm) and PTW (3 to 5 μm), were used to strengthen the Fe-based IDB matrix. To investigate the effect of the 2 materials on the performance of the Fe-based IDB matrix, each of the two toughening materials was added to FJT-A2 powder at various contents of 0, 1 wt%, 2 wt%, 3 wt%, 4 wt%, and 5 wt%, and then mixed for 10 h in a ball mill mixer. The mixed powder was loaded into graphite molds and then sintered. Three types of samples for bending strength, impact toughness, and impact wear resistance tests were prepared via hot-pressing sintering. Because the melting point of FJT-A2 is approximately 850 °C, in referencing [8,11,15] and considering the special requirements for percussive rotary drilling, the sintering parameters were set at a sintering temperature of 900 °C, a sintering pressure of 15 MPa, and a sintering holding time of 3 min. The bending strength samples were divided into those with diamonds and those without diamonds. The diamond grit mesh size was 40/50, and the volume fraction of the diamonds in the diamond-containing sample was 25%. The sample sizes are shown in Table 1.

### 2.2. Property Tests and Microscopic Analysis

**Figure 1:**
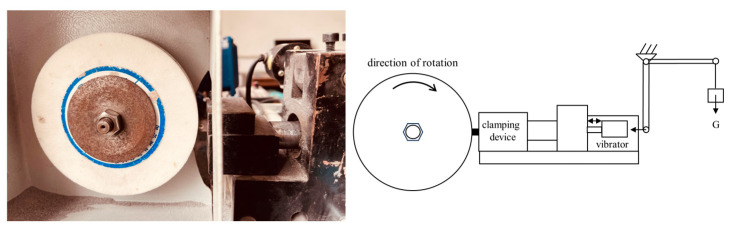
Impact wear test machine photograph and schematic image.

The hardness, bending strength, relative density, and wear resistance under impact conditions were used to evaluate the performances of the matrices. An HR-150A hardness tester was used to test the hardness of the samples. The three-point bending strength was tested using a computer-controlled universal material testing machine (CTM2500) with a span of 24.5 mm and loading speed of 20 N/s. A PTM2450 pendulum impact tester was used to measure impact toughness. The relative density in this study was defined as the ratio of the actual density of the matrix sample to its theoretical density, and its actual density was obtained using the Archimedes principle of buoyancy. Impact wear resistance testing was performed using a wear ratio tester (HMH-25) with a corundum grinding wheel (diameter: 125 mm; particle size: 80 US mesh; hardness L—moderate hardness). The impact was achieved using a mechanical vibrator (amplitude: 6 mm, frequency: 20 to 60 Hz, impact force: 1 to 18 kgf). Each test group was subjected to a load of 25 N (conforming to the pressure during drilling), a linear velocity of 4.2 m/s, and a wear time of 0.5 min. The impact energy in the experiment was set at 0.9 J by adjusting the position of the vibrator, and the impact frequency was 45 Hz. A schematic is shown in Figure 1. In this article, the impact tribological performance refers to the wear rate, which is the mass loss of the sample per minute (g/min). The fracture surfaces of the samples after the bending strength test and the surface morphology of the worn samples were analyzed via SEM, and the diamond on the fracture surface of the diamond-containing sample was tested via energy-dispersive X-ray spectroscopy. The surface roughness of the worn sample was also measured using a 3D laser scanning confocal microscope. The abrasive debris generated during the experiment was collected, and a sieving test and SEM observations were performed.

## 3. Results and Discussion

### 3.1. Mechanical Properties

**Figure 2:**
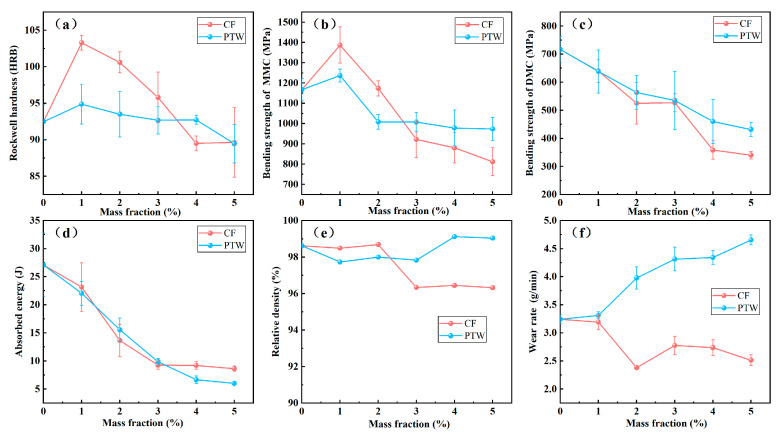
Relationships between sample mechanical properties and the content of CF and PTW: (**a**) hardness; (**b**) bending strength of MMC; (**c**) bending strength of DMC; (**d**) impact toughness; (**e**) relative density; (**f**) wear rate under impact condition.

Experimental results of the samples with various toughening material contents are shown in Figure 2. It can be seen that a small amount of CF and PTW had a positive effect on the Rockwell hardness (HRB) and bending strength of the blank matrix without diamonds. When the mass fraction of the two toughening materials was increased from 0 to 1 wt%, the hardness and bending strength of the pure matrix sample increased. When the content of the two materials was over 1 wt%, the hardness and bending strength began to gradually decrease with the increase in the content. As shown in Figure 2a, without adding the two toughening materials, the hardness of the sample was 92.5 HRB. At a 1 wt% content of toughening materials, the hardness value of the CF group increased by 11.71%, whereas that of the PTW group increased by 2.60%. Overall, the impact of the CF on matrix hardness exceeded that of the PTW. In Figure 2b, when the addition amount was 0, the bending strength of the metal matrix composite (MMC) was 1167 MPa. With the increase in the amounts of toughening materials, the bending strength of the MMC first increased and then decreased. When the mass fraction of the two added materials was 1 wt%, the bending strength of the two MMCs was at the maximum. The bending strengths of the CF and PTW groups were 1387 MPa and 1237 MPa, respectively, which were 18.85% and 6.00% higher, respectively, than those of the group without reinforcement. Figure 2c,d show that the bending strength and impact toughness of the diamond matrix composite (DMC) were adversely affected by an increase in material content. The difference was that for the MMC samples, the bending strength of the sample containing PTW was higher than that of the sample containing CF. As shown in Figure 2e, the relative density of most samples was above 97.70%, except for CF samples with a mass fraction higher than 3 wt%, but their relative densities were also higher than 96%. Obviously, both materials could strengthen an Fe-based IDB matrix, but CF had a better reinforcing effect.

**Figure 3:**
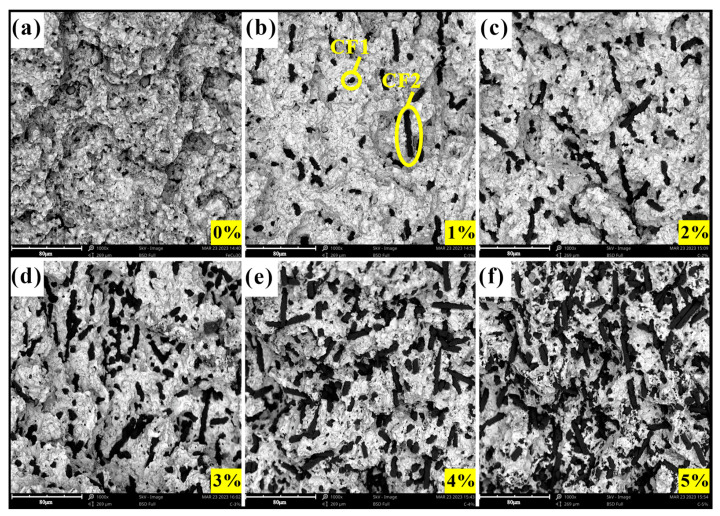
The fracture morphology of MMC: (**a**) 0 wt% CF; (**b**) 1 wt% CF; (**c**) 2 wt% CF; (**d**) 3 wt% CF; (**e**) 4 wt% CF; (**f**) 5 wt% CF.

**Figure 4:**
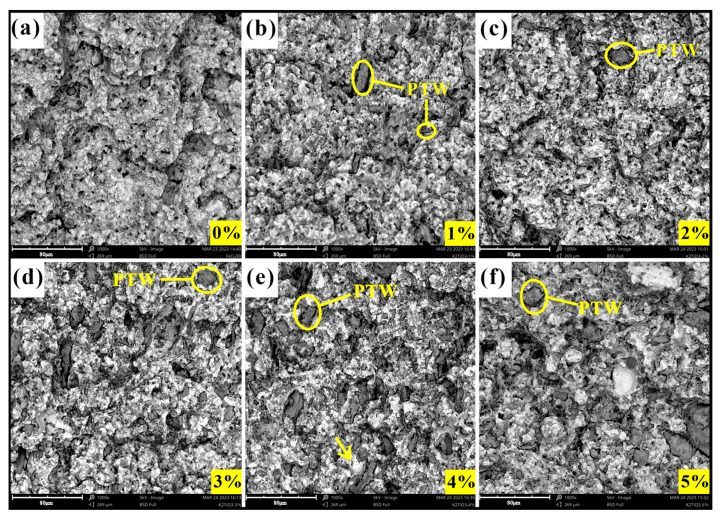
The fracture morphology of MMC: (**a**) 0 wt% PTW; (**b**) 1 wt% PTW; (**c**) 2 wt% PTW; (**d**) 3 wt% PTW; (**e**) 4 wt% PTW; (**f**) 5 wt% PTW.

SEM image results of the fracture surface after the bending strength test are shown in Figure 3 and Figure 4. It can be observed that there were more or fewer pores in the matrix, which was due to the hot-pressing sintering process. The increase in the content of added materials can be clearly observed in the figure. The distribution states of the CFs and PTWs in the matrix were also different. The CFs maintained their original state well and were completely embedded in the matrix, whereas the PTWs, due to their small size, underwent significant aggregation after mixing and sintering, and the bonding with the matrix was not too tight. Adding a small amount of CF and PTW increased the hardness of the matrix. Generally speaking, the smaller the grain size, the greater the strength of the interface [38]. Therefore, the increase in matrix hardness might have been due to the obstruction of grain size growth by the CF and PTWs. The fracture surfaces in Figure 3a–c and Figure 4a–c show that adding an appropriate amount of CF and PTW improved the grain uniformity of the matrix material. However, the hardness began to decrease as the content continued to increase. An excessive addition of materials reduced the uniformity of the grain size of the matrix, and there was a greater probability that the measurement point might fall on the added material during measurement.

**Figure 5:**
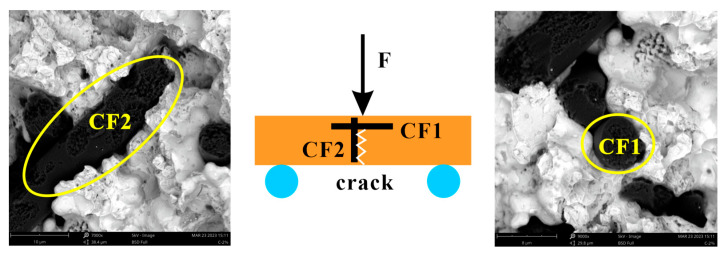
SEM micrographs showing the morphology of CF within the matrix.

The bending strength of the sample was influenced mainly by the bonding conditions between the materials and the relative density. For the same material, the higher the relative density, the stronger the material. When other materials were added, the strength of the composite related also to the properties of the newly added material, as well as the bonding strength between the interfaces of different materials. As shown in Figure 5, the three-point bending test is an experimental method for testing the mechanical properties of composites. The specimen was placed on two supporting cylindrical beams at a specified span of 24.5 mm, and a downward load at a loading speed of 20 N/s was applied to the specimen at the middle of the span. When the three contact points of the specimen formed two equal torques, three-point bending occurred, and the specimen fractured at the midpoint. Carbon fibers were distributed randomly in the sample, and states 1 and 2 were the two critical states that CFs exhibited in the matrix. There, CF1 was perpendicular to the fracture surface and the direction of the loading force, whereas CF2 was parallel to the fracture surface and the direction of the loading force. However, in fact, CFs in other directions of space could also decompose the loading force into forces perpendicular to and parallel to the CFs, and this type of CF-reinforced specimen with CFs perpendicular to the loading force had the strongest fracture resistance. For the loading conditions in Figure 5, when the specimen failed, there was not only the bonding force between the CFs and the matrix but also the failure strength of CF1. For CF2, only the bonding force existed between the CFs and the matrix. When external forces acted on the matrix, the CFs could reduce the stress concentration in the matrix by absorbing and dispersing the stress while also inhibiting crack propagation. When the CF content continued to increase, the CFs began to contact each other, reducing the bending strength of the MMC. A small amount of PTWs could be uniformly distributed in the matrix. Due to the different thermal expansion coefficients, residual stress was generated after sintering at the interface between the PTWs and the matrix. When external forces were exerted on the sample, cracks were generated, and they extended to the interface. The whiskers prevented further crack propagation through bridging, crack deflection, a whisker pull-out effect, and crystal breakage, thereby improving the bending strength of the MMC. However, when excessive PTW agglomerated, the bending strength of the MMC decreased.

**Figure 6:**
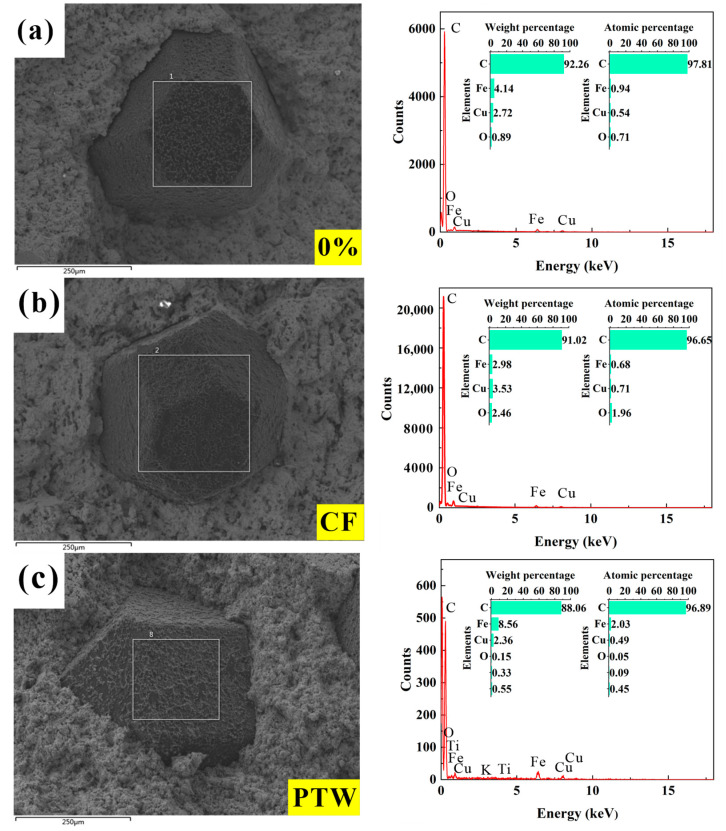
EDS analysis of DMC samples after bending strength test (The white frame is the scan area): (**a**) 100 wt% FJT-A2; (**b**) 1 wt% CF; (**c**) 2 wt% PTW.

**Figure 7:**
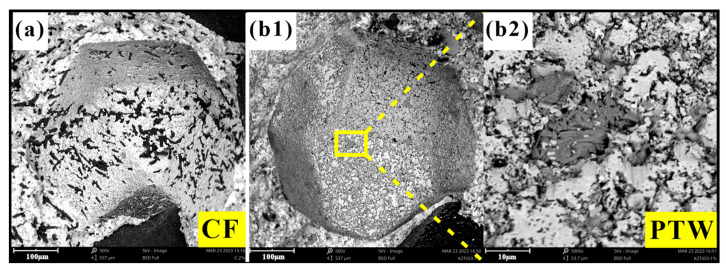
Morphologies of the diamond–metal interfaces: (**a**) CF; (**b1**) PTW; (**b2**) PTW.

The performance of the diamond-containing samples depended mainly on the holding force of the matrix on the diamond. Generally, the holding force of the matrix on the diamond consisted of mechanical embedding and chemical bonding forces. The holding capacity largely determined the bending strength of the DMC. Mechanical inlay occurred during the cooling process after hot-pressing sintering. Because the thermal expansion coefficient of the metal matrix was much higher than that of the diamonds, the matrix generated a compressive stress on the diamonds during the cooling process, ultimately forming residual stress on the diamond surfaces to effectively hold the diamonds. The chemical bonding force was generated by the chemical reaction between some components in the matrix and the contact interface of the diamonds during the hot-pressing sintering process. The interatomic force can effectively hold the diamonds. Energy-dispersive spectroscopy spot scanning was performed on the diamond surface on the fracture surface of the DMC; the results are shown in Figure 6. For the diamond surface of DMC samples containing CF, only C, Fe, Cu, and O elements were detected, while for the samples containing PTW, K and Ti were also detected, indicating that some matrix elements had infiltrated or diffused onto the diamond surfaces. However, on the whole, adding the two materials reduced the bending strength of the DMC, because the matrix’s ability to hold diamonds decreased. As shown in Figure 7, CF and PTW can be clearly observed at the interface between the diamonds and the matrix, which greatly weakened the mechanical embedding force of the matrix to a certain extent.

### 3.2. Impact Tribological Performance

#### 3.2.1. Impact Wear Rate

As shown in Figure 2f, the wear rate of the sample containing CF experienced a linear decrease followed by an increase and then a decrease, reaching the lowest value at 2 wt%. Overall, the impact wear rate of the sample with added CF was lower than that of the sample without added CF. The impact wear rate of the 100% FJT-A2 sample (without added fibers) was 3.24 g/min. After adding CF, the wear rate began to decrease, reaching a minimum value of 2.38 g/min at 2 wt%, which was 26.54% lower than that of the 100% FJT-A2 sample. When the CF content increased to more than 2 wt%, the wear rate increased but was still lower than the wear rate of the 100% FJT-A2 sample. Then, the wear rate continued to show a downward trend when the content was more than 3 wt%. As the content of CF increased to more than 2 wt%, the sample’s wear resistance increased, which was due to the enhanced mechanical strength of the MMC. As the content increased to more than 3 wt%, the mechanical properties of the MMC began to decrease and the wear rate began to increase. The CFs used in that experiment were graphitized, and as a self-lubricating material, graphite can improve the tribological properties of the MMC [39]. Therefore, we could observe the second critical point, where the wear rate of the MMC began to decrease for the second time at 3 wt%. This might have been because of the formation of a lubricating film on the sliding surface of the worn graphitized CF, which compensated for the negative effect of the decrease in mechanical strength. Therefore, it was speculated that there was a third critical point outside the range set in the experiment, where the decrease in mechanical strength exceeded the compensation of the lubricating film, and the wear rate continued to increase after that point. In contrast, the wear rate of the PTW samples continued to increase, indicating poor impact wear resistance.

#### 3.2.2. Impact Wear Surface Analysis

**Figure 8:**
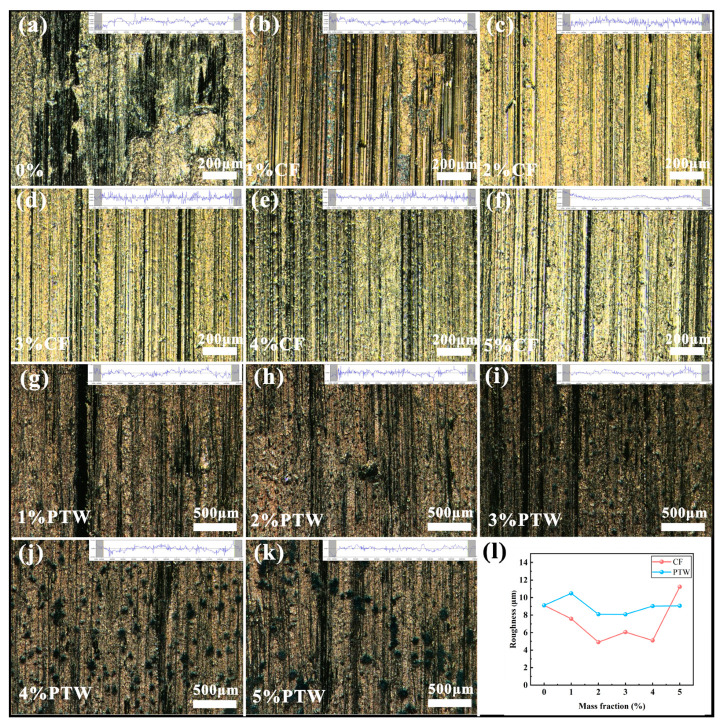
Optical topography and roughness of the worn surface: (**a**) 0 wt% CF; (**b**) 1 wt% CF; (**c**) 2 wt% CF; (**d**) 3 wt% CF; (**e**) 4 wt% CF; (**f**) 5 wt% CF; (**g**) 1 wt% PTW; (**h**) 2 wt% PTW; (**i**) 3 wt% PTW; (**j**) 4 wt% PTW; (**k**) 5 wt% PTW; (**l**) roughness.

The optical morphology of the worn sample surface is shown in Figure 8, and the cross-sectional profile perpendicular to the wear direction is in the upper right corner of the respective optical images in the illustrated mode. FJT-A2, as an iron–copper pre-alloyed material, is relatively soft. When it is worn against a grinding wheel, the contact is relatively tight under pressure and impact, so the sample heats very quickly. Figure 8a shows that there was obvious abrasive and adhesive wear on the surface. For CF samples, adding CF significantly changed the wear mechanism, and the surface of the sample had obvious wear grooves. On one hand, adding CF improved the hardness and bending strength of the IDB matrix, but on the other hand, the worn CF played a certain role in lubrication and reduced the friction temperature. The alteration in the roughness (Ra) of the worn surface is shown in Figure 8l. The change in surface roughness of the CF specimens was consistent with the wear rate, except for specimens with 5 wt% CF. The surface roughness of the CF samples with a content of 0 to 4 wt% first decreased, then increased, and then decreased. The minimum roughness was 4.91 μm measured on the 2 wt% CF sample. Compared to the roughness of the sample without the addition of CF, it decreased by 46.16%, but the roughness of the 5 wt% CF sample increased sharply, which might have been due to the wide distribution of CFs, as shown in Figure 3f. For the PTW sample, the wear surface is significantly different from the CF wear surface, and the wear groove was not as uniform as in the CF sample. Adding PTW did not affect its surface roughness as much as CF. The Ra value of the worn surface of the PTW sample decreased by a maximum of 11.31% compared to the sample without PTW. However, the Ra values of all samples fluctuated within 1 μm, and overall, the surface roughness values of the PTW samples exceeded those of the CF samples.

**Figure 9:**
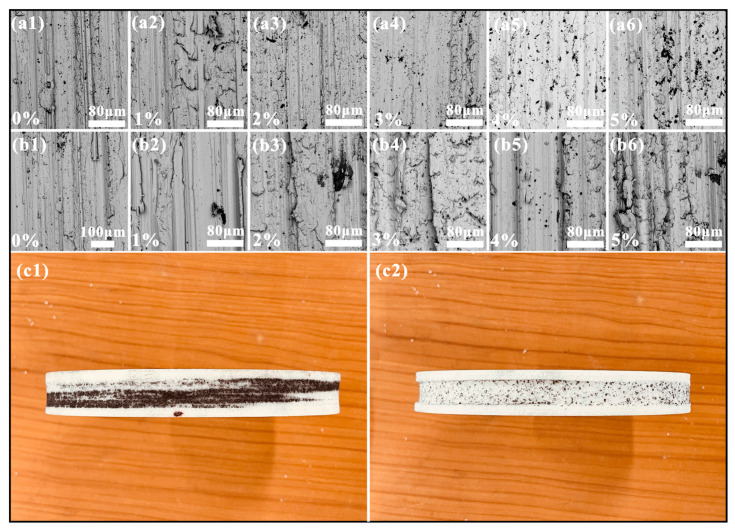
SEM micrographs of the worn surface and the corundum grinding wheel: (**a1**) 0 wt% CF; (**a2**) 1 wt% CF; (**a3**) 2 wt% CF; (**a4**) 3 wt% CF; (**a5**) 4 wt% CF; (**a6**) 5 wt% CF; (**b1**) 0 wt% PTW; (**b2**) 1 wt% PTW; (**b3**) 2 wt% PTW; (**b4**) 3 wt% PTW; (**b5**) 4 wt% PTW; (**b6**) 5 wt% PTW; (**c1**) corundum grinding wheel after impact wear test with samples (CF); (**c2**) corundum grinding wheel after impact wear test with samples (PTW).

To further explore the impact wear mechanism of the MMC, a SEM analysis was performed on its worn surface; the results are shown in Figure 9. At the microscopic level, the grooves on the surface of the sample can still be seen. Under the influence of impact, some abrasive particles are deeply embedded in the matrix, forming obvious wear marks. Due to the squeezing and pulling of abrasive particles, plastic deformation occurred on both sides of the wear marks, making it easier for the matrix to peel off. The surfaces of the CF samples were relatively smooth, with smaller and shallower wear marks. This indicates a decrease in abrasive wear on the friction surface. As shown in Figure 9a6, the number of CFs on the worn surface increased as the CF content increased. Due to the adhesive nature of graphite, it adhered to the friction surface. As shown in Figure 9c1, as wear continued, it could be observed that more and more graphite adhered to the surface of the corundum grinding wheel, forming a black lubricating film on its surface that effectively reduced the wear rate of the MMC. However, the PTW samples had not only abrasive wear but also a significant amount of adhesive wear. In Figure 9c1, it can be observed that there was obvious metal adhesion on the surface of the corundum grinding wheel. Moreover, the grooves on the worn surface were deeper and wider because of their low hardness, making it easier for abrasive particles to penetrate, and the friction surface was severely damaged under the effects of temperature and shear force. A convex area formed on both sides of the large pulling groove. As wear progressed, the convex area was squeezed and worn, resulting in peeling, which increased the wear rate.

#### 3.2.3. Debris Analysis

**Figure 10:**
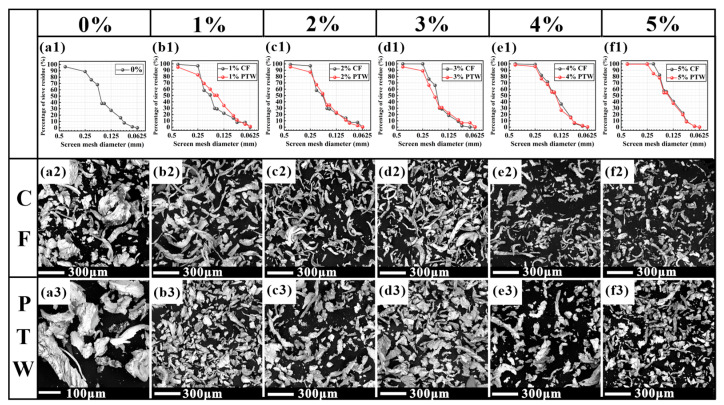
Abrasive particle gradation curves (first line) and SEM micrographs of the abrasive debris (the second and third lines): (**a1**) FJT-A2; (**b1**) 1 wt% CF and PTW; (**c1**) 2 wt% CF and PTW; (**d1**) 3 wt% CF and PTW; (**e1**) 4 wt% CF and PTW; (**f1**) 5 wt% CF and PTW; (**a2**) 0 wt% CF; (**b2**) 1 wt% CF; (**c2**) 2 wt% CF; (**d2**) 3 wt% CF; (**e2**) 4 wt% CF; (**f2**) 5 wt% CF; (**a3**) 0 wt% PTW; (**b3**) 1 wt% PTW; (**c3**) 2 wt% PTW; (**d3**) 3 wt% PTW; (**e3**) 4 wt% PTW; (**f3**) 5 wt% PTW.

A magnet was used to distinguish the worn sample debris from the grinding wheel abrasive, and the abrasive debris was analyzed. A sieving test was performed on the abrasive debris, and the abrasive particle gradation curves are shown in Figure 10. Through the sieving test, it was found that the maximum particle size of the abrasive debris in the CF sample was smaller than that in the PTW sample. The maximum screen size used for the test was 40 mesh, through which the abrasive debris of the CF sample could pass, while the abrasive debris of the FJT-A2 and PTW samples could not pass. The average size of the abrasive debris of the CF samples was larger than that of the PTW samples when the material additions were 1 wt% and 2 wt%, while the results were just the opposite at other addition amounts. It was difficult to visualize the impact wear performance of the samples from the abrasive particle gradation curves, so a SEM analysis was performed on all abrasive debris. For the FJT-A2 sample, there were predominantly large chips as well as a few strips of chips, which corresponded to the adhesive and abrasive wearing described in Section 3.2.2. During the sample’s wear process, the interaction mechanism between the abrasive particles and the matrix became more complex due to the rotation of the grinding wheel and the periodic impact force. In the grinding area, the grinding process could be divided into scratch and plowing processes. When an impact was generated, the cutting depth between the grinding wheel particles and the matrix instantly increased, and the plowing effect dominated. The hardness and other properties of the material itself also determined the wear mechanism. Compared to PTW debris, it was evident that the abrasive debris of the CF sample was mainly continuous serrated chips, whereas the abrasive debris of the PTW sample was mainly banded chips.

**Figure 11:**
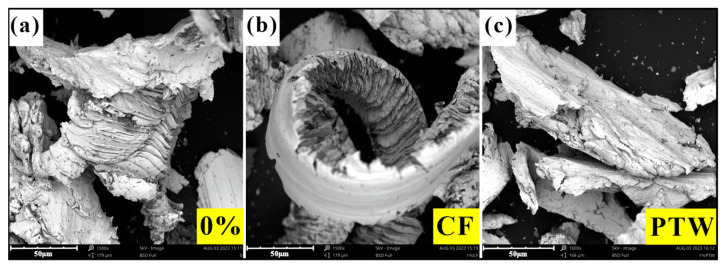
SEM micrographs of the abrasive debris: (**a**) FJT-A2; (**b**) 1 wt% CF; (**c**) 1 wt% PTW.

When the abrasive particles of the grinding wheel were pressed into the matrix under pressure, as they rotated, the matrix underwent shear slip under the compression of the abrasive particles. The matrix directly below the abrasive particles was extruded into chips, and plastic deformation occurred on both sides. The wear debris generated by impact wear consisted mainly of serrated and banded shapes. Figure 11 shows the typical characteristics of abrasive debris under three formulations. The abrasive debris thickness of the CF sample was significantly lower and corresponded to the CF’s low impact wear rate. The serration of abrasive debris and an increase in cutting depth would increase the frequency of abrasive debris breakage. As shown in Figure 9, the worn surface of the PTW sample had deeper furrows, and when wear occurred, excessively thick chips were more likely to form banded debris under impact.

## 4. Conclusions

The matrix’s performance largely determines the IDB’s life, especially during percussive–rotary drilling, where the force on the bit is more complex than in other types of drilling. An excellent performance of the IDB matrix can effectively improve the drilling efficiency and bit life, resulting in lower drilling costs. In this article, the effects of CF and PTW on the mechanical and impact tribological properties of an Fe-based IDB matrix were studied. Based on the above results and discussion, the following conclusions could be drawn:

(1) The hardness and bending strength of the MMC were strengthened by adding proper amounts of CF and PTW into an Fe-based IDB matrix, and the best performance was achieved when the addition was 1 wt%. The strengthening effect of CF exceeds that of PTW.
(2) Carbon fiber and PTW play weakening roles in the bending strength of DMCs and the impact toughness of the matrix.
(3) Carbon fiber significantly strengthens the impact tribological properties of an Fe-based IDB matrix, with the lowest wear rate at a 1 wt% dose. However, PTWs weaken the impact wear resistance of the Fe-based IDB matrix, which is opposite to the effect of CFs.
(4) In this study, the condition of the wear surface of the CF sample was significantly improved, and a lubricating film was formed during the wear process, resulting in a significant reduction in surface roughness, reaching a minimum of 4.91 μm. Compared to the 100% FJT-A2 sample, the surface roughness decreased by 46.16%. By contrast, the worn surface of the PTW sample was clearly grooved, indicating poor impact wear resistance.
(5) The abrasive debris of the CF sample was mainly serrated and continuous, whereas that of the PTW sample was mainly banded and discontinuous.

Overall, for this study, CF as a reinforcing material outperformed PTW in terms of the mechanical and tribological properties of an Fe-based IDB matrix. Carbon fiber can be used as a reinforcing material for the IDB matrix used in percussive–rotary drilling. As for PTWs, due to their weak impact wear resistance, they can be applied to “slipping” formations, where diamond bits self-sharpen only with difficulty.

## Figures and Tables

**Table 1:** Sample dimensions for different tests.

Sample	Hardness/Impact Wear	Bending Strength	Impact Toughness
Dimensions (mm^3^)	8.5 × 8.5 × 15	5 × 5 × 30	10 × 10 × 55

## Data Availability

Data are contained within the article.
